# Bioactive Adrenomedullin in Dogs with Sepsis and Septic Shock: A Prospective, Case-Control Study

**DOI:** 10.3390/ani14213054

**Published:** 2024-10-23

**Authors:** Eirini Chrysovergi, Carmel T. Mooney, Robert E. Shiel, Evangelia M. Stavroulaki, Kevin Murtagh

**Affiliations:** 1 University College Dublin (UCD) Veterinary Hospital, University College Dublin, D04 Dublin, Ireland; carmel.mooney@ucd.ie (C.T.M.); kevin.murtagh@ucd.ie (K.M.); 2 School of Veterinary Medicine, Murdoch University, Perth 6000, Western Australia, Australia; robert.shiel@murdoch.edu.au; 3 DWR Veterinary Specialists, Six Mile Bottom CB8, UK; eva_stavroulaki@yahoo.gr

**Keywords:** adrenomedullin, biomarker, disease severity

## Abstract

Sepsis and septic shock are leading causes of morbidity and mortality in both humans and dogs, and early diagnosis is crucial for improving outcomes. This study aimed to evaluate bioactive adrenomedullin (bio-ADM) concentrations in dogs with septic shock (n = 25), dogs with sepsis without evidence of shock (n = 25), and healthy control dogs (n = 25). Plasma bio-ADM concentrations were measured using a human sandwich enzyme-linked immunosorbent assay and reported as median (interquartile range). Plasma bio-ADM concentrations were significantly higher in both septic groups compared to the healthy controls (all <22.4 pg/mL), but not significantly different between the septic shock (75.0 [28.7–115.0] pg/mL) and sepsis (30.7 [22.4–79.7] pg/mL) groups. Dogs with higher illness severity scores had significantly higher bio-ADM concentrations (93.1 [32.2–122.0] pg/mL) than those with lower scores (29.8 [22.4–71.2] pg/mL). However, bio-ADM concentrations did not differ between survivors (33.0 [22.7–76.7] pg/mL) and non-survivors (74.7 [26.1–123.2] pg/mL). Measurement of bio-ADM is a potential marker for canine sepsis, but not for the identification of septic shock, and may provide information on disease severity. Further studies, including those on non-infectious inflammatory conditions, are necessary to better understand the diagnostic utility of bio-ADM measurement and its potential role as a marker of treatment response in dogs with sepsis.

## Simple Summary

Sepsis and septic shock are life-threatening conditions affecting both humans and dogs. Early detection and treatment are crucial for improving outcomes, but timely diagnosis remains challenging. Adrenomedullin (ADM), a naturally occurring hormone with a potent vasodilatory effect, has emerged as a potential biomarker to aid in the diagnosis and prognostication of sepsis and septic shock in humans. This study investigated the utility of the active form of this hormone, bioactive ADM (bio-ADM), in dogs. The study found that circulating bio-ADM concentrations were higher in both septic groups compared to healthy dogs, indicating its potential as a diagnostic tool for canine sepsis. However, there was no difference in bio-ADM concentrations between dogs with sepsis and those with septic shock. The study also explored the possibility of using bio-ADM as a predictor of disease severity and prognosis. While bio-ADM concentrations were associated with more severe illness, they did not predict survival. More research is needed to fully understand the role of bio-ADM in sepsis and its potential value in improving treatment strategies for dogs.

## 1. Introduction

Sepsis and septic shock are major health problems in both human and veterinary medicine [1]. Despite significant advances in understanding their pathophysiology, morbidity and mortality rates remain high in both humans and dogs [2,3]. Timely diagnosis and accurate prognostication remain challenging [4,5]. If not promptly treated, sepsis can rapidly escalate to severe sepsis or septic shock, leading to multi-organ failure and death [6]. Early recognition of sepsis can facilitate timely treatment with broad-spectrum antimicrobials, fluid resuscitation, and vasopressor support [7]. This can help stabilize hemodynamic parameters and significantly reduce pathogen load, ameliorating the systemic inflammatory response and subsequent organ dysfunction, which in turn shortens hospital stays, reduces complications, and ultimately improves survival [8,9,10]. The identification of a non-invasive, reliable biomarker capable of aiding the early diagnosis and risk stratification of sepsis in dogs would be advantageous for guiding treatment and improving outcomes.

Adrenomedullin (ADM) is a 52-amino acid peptide hormone that was originally isolated from pheochromocytoma cells and has emerged as a promising biomarker in the diagnosis of sepsis in humans [11]. Adrenomedullin has pleiotropic effects including vasodilation and stabilization of the endothelial barrier, which consequently reduces vascular permeability, lowers arterial pressure, increases heart rate and cardiac output, and maintains organ perfusion [12,13]. Beyond its vascular effects, ADM exhibits immunomodulatory, cardioprotective, anti-inflammatory, and bactericidal properties, emphasizing its importance in the pathophysiology of sepsis and septic shock [14,15,16].

Adrenomedullin has intermediate, inactive forms and a mature, active form known as bioactive ADM (bio-ADM) [17]. Numerous human studies have demonstrated the clinical utility of both the active and inactive forms of ADM as diagnostic and prognostic biomarkers in sepsis. Circulating ADM concentrations have been reported to be significantly higher in human patients with septic shock compared to controls, with increased ADM concentrations correlating with disease severity, higher mortality rates, and organ dysfunction [18,19,20,21]. Furthermore, ADM concentrations have been correlated with the extent of vasodilation and vasopressor requirements [20,22,23,24].

Inactive ADM can be measured in various ways including radioimmunoassays, enzyme immunoassays, liquid chromatography–mass spectrometry, and immunohistochemistry [25,26,27,28]. More recently, an non-automated immunoluminometric assay (ILMA) has been developed for the measurement of bio-ADM, by including antibodies against the amidated C-terminus as well as the mid-portion of the molecule [29].

In veterinary medicine, ADM concentrations have been shown to significantly increase in dogs with experimental congestive heart failure, and to correlate with the stage of heart disease [30,31,32]. Increased concentrations were also observed in dogs with hemorrhagic shock [33]. These studies measured ADM using radioimmunoassays that detect a combination of both inactive and active hormones. However, measurement of the active hormone is likely to correlate more accurately with intrinsic biological activity compared to non-selective adrenomedullin molecules [29]. An IMLA for the measurement of human bio-ADM (Sphingotec GmbH, Hennigsdorf, Germany) is commercially available and is reported to have 100% cross-reactivity with the synthetic canine ADM peptide [29]. Despite its promise as a diagnostic marker of sepsis and septic shock in humans, there are no studies to date evaluating the utility of bio-ADM measurement in dogs.

The primary aim of this study was to prospectively compare plasma concentrations of bio-ADM in healthy dogs, dogs with septic shock, and dogs with sepsis without signs of shock. It was hypothesized that the bio-ADM concentration would be higher in the septic and septic shock dogs when compared to the healthy animals, and that bio-ADM would be higher in the septic shock group when compared to the septic group. A secondary aim was to determine if bio-ADM could be prognostic in dogs with sepsis.

## 2. Materials and Methods

### 2.1. Study Population

This was a prospective, case–control study composed of client-owned dogs that were referred to the University College Dublin Veterinary Hospital for assessment and management of either sepsis or septic shock between January 2021 and March 2024. Residual blood samples were used providing sufficient volume remained after the primary diagnostic tests were completed.

Dogs were classified into three groups: dogs with septic shock (SS), dogs with sepsis without evidence of shock (SE), and healthy (control) dogs. The diagnosis of sepsis was based on documentation of a source of bacterial infection based on clinicopathological, cytological, and/or microbiological evidence. In cases of aspiration pneumonia, where obtaining a sample for cytology or microbiology posed a risk to the dog, supportive imaging findings and a high index of suspicion based on clinical signs were required. In addition to the evidence of infection, dogs needed to fulfill at least two criteria for systemic inflammatory response syndrome (SIRS) [34]. The following SIRS criteria were used in the study: rectal temperature less than 38.1 °C or greater than 39.2 °C; heart rate greater than 120/min; respiratory rate greater than 20/min; WBC less than 6 × 10^9^/L or greater than 16 × 10^9^/L or greater than 3% band neutrophils [34]. Septic shock was diagnosed following the documentation of sepsis with concurrent persistent hypotension, despite adequate fluid resuscitation, requiring vasopressor support to maintain blood pressure. Hypotension was defined as systolic blood pressure (SBP) < 90 mmHg or mean arterial pressure (MAP) < 60 mmHg. Blood pressure was obtained via direct or indirect methods (i.e., peripheral arterial catheter, Doppler, or oscillometric). Blood pressure measurement was a requirement in all SS dogs and although routine for dogs in the SE group, it was not assessed if not deemed clinically indicated by the primary clinician. The control group consisted of clinically healthy blood donor dogs.

Hematology and biochemistry analyses were performed for all dogs. The hematology was performed using either ADVIA 2120 (Siemens Healthcare Diagnostics, Ashburn, Germany) or IDEXX ProCyte Dx (IDEXX Laboratories, Westbrook, ME, USA). Biochemistry was performed using either Atellica CH (Siemens Healthcare Diagnostics, Ashburn, Germany) or IDEXX Catalyst One (IDEXX Laboratories, Westbrook, ME, USA). Analyzer use was predicated by sample acquisition timing (routine or out-of-hours). Additional diagnostic tests for the SS and SE were conducted at the discretion of the primary clinician (e.g., blood gas analysis, urinalysis, urine culture, diagnostic imaging (ultrasound, radiography, CT, or MRI), cytological or microbiological testing). Blood gas analysis was carried out using the Rapidpoint 500 (Siemens Healthcare Diagnostics, Ashburn, Germany). All dogs in the SS and SE groups received antimicrobials. The choice of antimicrobial was based on culture and susceptibility results or based on the clinician’s preference where such results were not available. The administration of antimicrobials prior to referral was not considered an exclusion criterion.

Data collected included signalment, body weight, relevant hematology and biochemistry results, blood gas analyses, baseline blood pressure, vasopressor requirements, final diagnosis, and outcome. Outcome was defined as either survival to discharge or death (encompassing both natural death and euthanasia). The severity of illness was retrospectively assessed using data recorded on the day the sample was collected. This assessment utilized the canine acute patient physiologic and laboratory evaluation (APPLE_fast_) score, which integrates clinical (i.e., mentation score (5-point scale)) and laboratory abnormalities (i.e., platelet count (5-point scale), glucose (5-point scale), albumin (5-point scale), and lactate (4-point scale) concentrations) into a composite score with a maximum value of 50 [35]. A cutoff value of ≥25, with a reported specificity of 85% for predicting mortality in dogs, was used [35]. The APPLE_fast_ score was calculated based on data recorded within the case records contemporaneous with data used for categorization and timing of sample collection.

### 2.2. Bioactive Adrenomedullin Measurement

Residual plasma samples were prepared by centrifugation of blood collected in ethylenediaminetetraacetic acid (EDTA) tubes (Sarstedt, Germany). Following centrifugation, the samples were stored at −80 °C and subsequently shipped on dry ice to an external commercial laboratory (Sphingotec GmbH, Germany) for batch measurements of plasma bio-ADM concentrations using a human heterologous sandwich IMLA (Sphingotec GmbH, Hennigsdorf, Germany) [29]. All samples were run in duplicate, following the laboratory protocol, and the mean value was reported.

The working range of the assay was 22.4 pg/mL to 1094.0 pg/mL with a manufacturer-reported human reference interval of ≤29 pg/mL. Intra-assay precisions (coefficient of variation (CV)) of 10 replicates of canine samples at low (<22.4 pg/mL), medium (67.0 ± 3.5 pg/mL), and high (885.2 ± 39.3 pg/mL) concentrations were 0.0%, 5.3%, and 4.4%, respectively. Inter-assay precision across two separate runs was 0.0% at <22.4 pg/mL, 2.7% at 56.1 ± 1.5 pg/mL, and 4.6% at 159.2 ± 7.3 pg/mL. There was no significant difference (*p* = 0.97) in bio-ADM concentrations following a freeze–thaw cycle (74.00 (44.5–118.4) pg/mL versus 79.3 (40.0–131.4) pg/mL). There was no significant difference (*p* > 0.05) in measured concentrations in samples rendered icteric, lipemic, or hemolytic.

### 2.3. Statistical Analysis

A power analysis was performed to calculate the number of dogs required to detect a significant difference in plasma bio-ADM concentrations (at the 5% level), between dogs with sepsis, with and without shock, and control dogs, with 90% statistical power. This analysis was based on preliminary data from 20 dogs, which included 10 control dogs and 10 dogs with septic shock. The bio-ADM range in non-healthy, non-septic dogs is largely unknown, as is the percentage of hospitalized dogs that develop sepsis. In light of these limitations, a conservative approach was used for sample size calculation, i.e., 50% as an effect size, 95% as a confidence level, and 20% as relative standard error. Results from these estimations indicated that 25 dogs should be included in each group [36].

Normality of continuous data was tested by visual inspection of the data and by using the Shapiro–Wilk test. Results are presented as mean ± standard deviation (sd) or median (interquartile range; IQR), as appropriate. Comparisons between groups were performed using Student’s t-test, Mann–Whitney test, or Kruskal–Wallis with Dunn’s post hoc analysis for multiple comparisons, as required. Differences in categorical data between groups were identified using the Chi-square test (or Fisher’s exact test when appropriate). Statistical significance was set at *p* < 0.05.

All samples with a bio-ADM concentration <22.4 pg/mL were assigned a value of 22.4 pg/mL for statistical analyses. All analyses were performed using statistical software packages (SPSS 23 for Windows IBM Corp., Armonk, NY, USA; and Prism 9, GraphPad Software Inc., Boston, MA, USA).

## 3. Results

### 3.1. Cases

In total, 75 dogs were included with 25 in each group (SS, SE, and control). The summary characteristics for each group are displayed in Table 1.

Breeds represented were mixed breed (n = 10), Labrador retriever (n = 10), golden retriever (n = 10), French bulldog (n = 5), greyhound (n = 4), German shepherd dog (n = 3), collie (n = 3), Bichon Frise (n = 3), Weimaraner (n = 2), miniature schnauzer (n = 2), Tibetan terrier (n = 2), cocker spaniel (n = 2), lurcher (n = 2), Bernese mountain dog (n = 2), and one each of the following breeds: bearded collie, Belgian shepherd dog, boxer, Hungarian vizsla, Irish wolfhound, Jack Russell terrier, Old English sheepdog, Patterdale terrier, German pointer, Pomeranian, Rottweiler, Samoyed, Scottish deerhound, Springer spaniel, and Saint Bernard.

There were no significant differences in sex (*p* = 0.686) or neuter status (*p* = 0.433) between the groups. Age and body weight were significantly different (*p* = 0.02 and <0.001, respectively). Dogs in the control group were significantly (*p* = 0.017) younger compared to those in the SE group and dogs in the control group were significantly heavier than those in both the SS (*p* = 0.009) and SE (*p* < 0.001) groups. Scores for APPLE_fast_ were significantly (*p* = 0.007) higher in the SS group compared to the SE group. Outcome was significantly different (*p* = 0.046) between the SS and SE groups.

The diagnoses in the SS group were as follows: septic peritonitis (n = 14, 56.0%), hepatobiliary infection (n = 3, 12.0%), pneumonia (bronchopneumonia or aspiration pneumonia) (n = 3, 12.0%), and one (4.0%) each of pyelonephritis, emphysematous cystitis, pyothorax, acute hemorrhagic diarrhea syndrome with associated septic shock, suspected to be due to bacterial translocation, and severe phlebitis.

In the SE group, the diagnoses were as follows: pneumonia (n = 9, 36.0%), septic peritonitis (n = 6, 24.0%), pyometra (n = 3, 12.0%), discospondylitis (n = 2, 8.0%), and one (4.0%) of bacterial prostatitis/endocarditis, pyelonephritis, cholecystitis, osteomyelitis, and ovarian pedicle abscess.

### 3.2. Comparison of bio-ADM Concentrations

**Figure 1:**
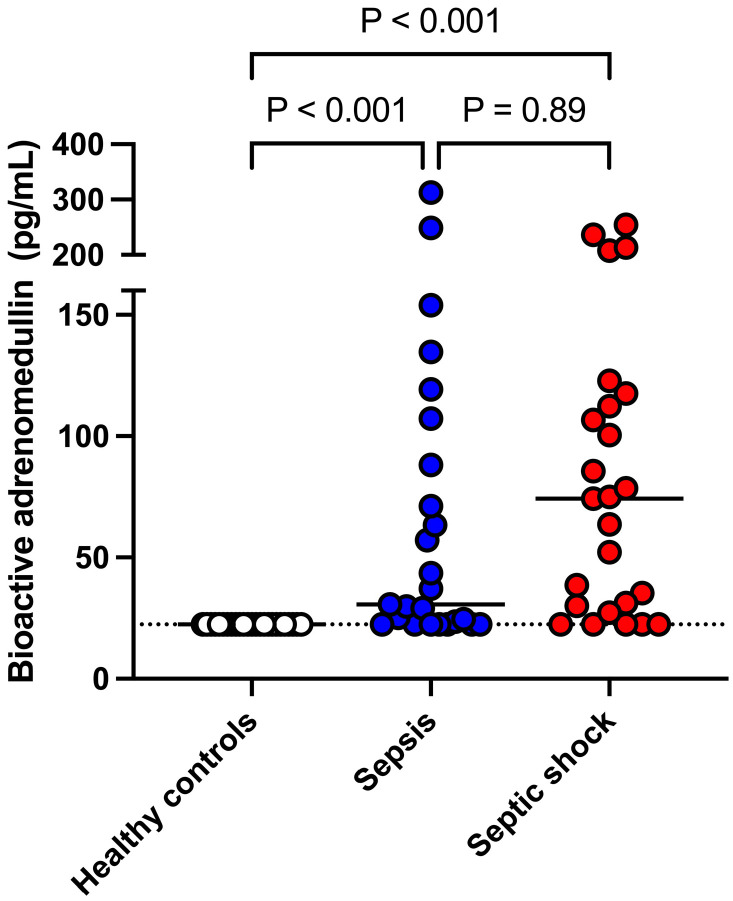
Dot-plot illustrating bioactive adrenomedullin concentrations in healthy dogs, dogs with sepsis, and dogs with septic shock. The solid line represents the median in each group. The dotted line represents the lowest measurable concentration of the assay (22.4 pg/mL).

The median bio-ADM concentrations in the SS (75 [28.7–115.0] pg/mL) and SE (30.7 [22.4–79.7] pg/mL) groups were significantly (*p* < 0.001, respectively) higher than in the control group (all <22.4 pg/mL) (Figure 1). However, there was no statistical difference (*p* = 0.89) in the bio-ADM concentration between the SS and SE groups.

**Figure 2:**
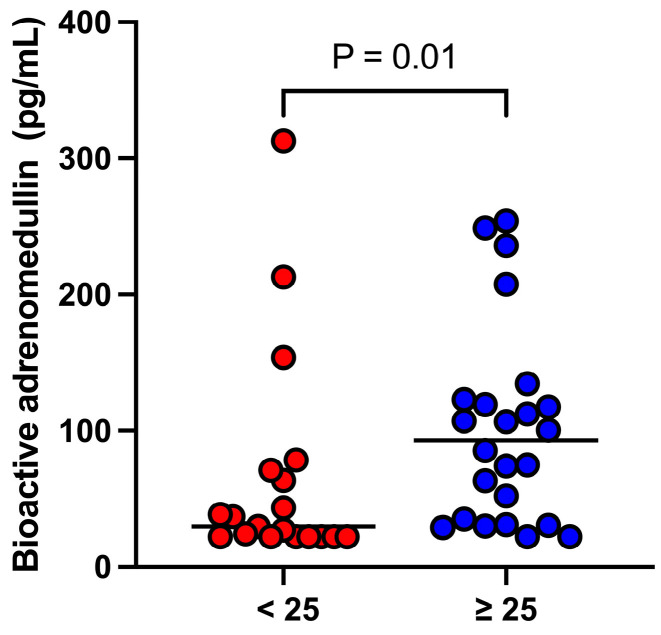
Dot-plot illustrating the bioactive adrenomedullin concentrations in relation to an acute patient physiologic and laboratory evaluation fast score of < or ≥25 in 43 dogs with septic shock (n = 25) or sepsis (n = 18). The solid line represents the median in each group.

The median bio-ADM concentration was significantly (*p* = 0.01) higher in dogs with APPLE_fast_ scores ≥ 25 (93.1 [32.2–122.0] pg/mL) compared to those with scores < 25 (29.8 [22.4–71.2] pg/mL (Figure 2).

**Figure 3:**
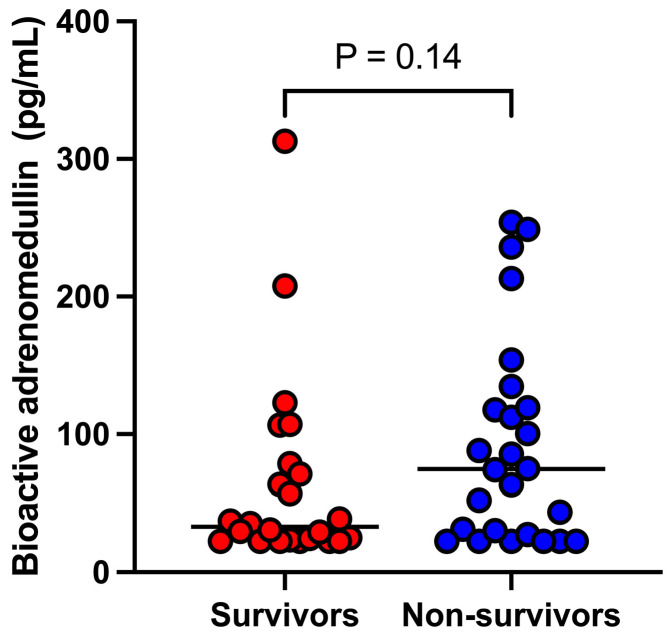
Dot-plot illustrating the bioactive adrenomedullin concentrations in dogs that did (n = 24) or did not (n = 26) survive.

The median bio-ADM concentration did not differ significantly (*p* = 0.14) between survivors (33.0 [22.7–76.7] pg/mL) and non-survivors (74.7 [26.1–123.2] pg/mL) (Figure 3).

In the SS group, nine (36.0%) had bio-ADM values > 100 pg/mL, with four dogs (16.0%) having values that exceeded 200 pg/mL. In the SE group, six dogs (24%) had bio-ADM values > 100 pg/mL, with two dogs (8%) having values exceeding 200 pg/mL. There was no significant difference (*p* = 0.544) in the proportion of survivors in dogs with a bio-ADM concentration < or >100 pg/mL.

## 4. Discussion

To the best of the authors’ knowledge, this is the first study describing bio-ADM concentrations in dogs. The study included dogs with septic shock, dogs with sepsis without shock, and a healthy control population, showing that both septic groups had significantly higher concentrations of bio-ADM compared to healthy dogs. This suggests that bio-ADM is a promising biomarker in the diagnosis of sepsis in dogs. However, no significant difference in bio-ADM concentrations was observed between dogs with septic shock and those with sepsis, without shock, indicating the need for further investigation into the factors influencing bio-ADM concentrations in these conditions.

One possible explanation for the lack of a significant difference in the bio-ADM concentration between the two septic groups could be that both groups included dogs with similar illnesses, with the primary distinction being the requirement for vasopressor therapy. The SE group, in particular, was notably heterogeneous, comprising some dogs that were critically ill and met multiple SIRS criteria but did not meet the criteria for septic shock, while others had a localized infection with no overt systemic involvement. Vasopressor requirement was used to distinguish the two septic groups in this study, but this requirement likely does not adequately differentiate the degree of inflammation seen between the groups. Therefore, some dogs within the SE group may not have required a vasopressor but could have had a similarly severe systemic disease, allowing for comparable bio-ADM concentrations between the two septic groups. Another explanation could be that different diseases, or even the same disease within individuals and at different timepoints, may be associated with varying severity of infection and/or inflammation, and therefore different bio-ADM concentrations. Several examples of this were seen in dogs in this study. For instance, dogs diagnosed with aspiration pneumonia had bio-ADM values ranging from <22.4 pg/mL to 154 pg/mL, and dogs with septic peritonitis had bio-ADM values ranging from <22.4 pg/mL to 213.1 pg/mL. The variability in bio-ADM concentrations in dogs with the same diagnosis suggests that bio-ADM concentrations may not correlate with disease type alone but may reflect the severity of systemic inflammation or infection. Lastly, many of the dogs in the septic groups were critically ill, and as all were referral patients, they were likely to have received treatments, such as fluid therapy and antimicrobials, prior to sample collection, which could have impacted their bio-ADM concentrations.

In the SS group, approximately one-third of dogs had bio-ADM values exceeding 100 pg/mL, with approximately half of these having values above 200 pg/mL. Uniformly high bio-ADM concentrations were expected within this group; however, many SS dogs had values below 100 pg/mL, with some even being undetectable. Similarly, in the SE group, about one-quarter of dogs had bio-ADM values exceeding 100 pg/mL, with a small percentage having values above 200 pg/mL. Interestingly, the proportion of dogs with bio-ADM concentrations exceeding these thresholds was similar between the SS and SE groups, which was also unexpected. As discussed above, this discrepancy may be due to the fact that some SE dogs in our study were very ill, leading to increased bio-ADM concentrations. Conversely, some SS dogs had lower-than-expected bio-ADM concentrations, possibly due to treatments they received prior to referral.

The APPLE_fast_ score was retrospectively applied to 43 dogs, based on hospital records at the time of sample collection, in both septic groups to objectively assess disease severity. This study demonstrated that the bio-ADM concentrations were significantly higher in dogs with APPLE_fast_ scores ≥ 25 compared to those with scores below 25. The observed difference indicates that bio-ADM could potentially serve as an indicator of disease severity in septic dogs. However, eight dogs died despite having low APPLE_fast_ scores, while seven dogs with high APPLE_fast_ scores survived. Survival of some dogs despite a high APPLE_fast_ score could reflect the timely and appropriate management of cases with treatable conditions, such as pyelonephritis, aspiration pneumonia, septic peritonitis, and pyometra. Non-survival of septic cases despite a low APPLE_fast_ score could be because this value was based on initial presenting data. Disease progression would result in a higher value, but serial APPLE_fast_ scores were not evaluated in this study. Therefore, a low APPLE_fast_ score assigned at the time of presentation may be an underrepresentation of the eventual severity of illness in some dogs, considering the dynamic nature of sepsis and septic shock. For instance, scores may have been higher shortly before death or euthanasia compared to at the time of admission, highlighting the need for serial evaluations to monitor clinical deterioration.

Interestingly, no significant difference in bio-ADM concentrations was observed between survivors and non-survivors, which suggests that while bio-ADM levels may be reflective of disease severity, they do not necessarily predict outcomes. This finding highlights the complexity of sepsis in dogs and suggests that bio-ADM, although a marker of systemic inflammation, may need to be considered alongside other clinical and diagnostic factors when assessing prognosis. Further research is needed to explore whether combining bio-ADM with other biomarkers or clinical scores could improve its prognostic utility. Additionally, as sample sizes for the determination of outcome were different, the statistical power may not have been adequate. This could have impacted the ability to detect significant differences in bio-ADM concentrations between survivors and non-survivors. Larger studies are required to confirm these findings.

Finally, differences in age and body weight between the septic and control groups were noted, with younger, larger dogs comprising the control group. This discrepancy is explained by the fact that control dogs were sourced from a blood donation program. Nevertheless, bio-ADM concentrations in all control dogs were undetectable, indicating that age and body size were not significant factors affecting bio-ADM concentrations in this study.

Our study had several limitations. Firstly, there was the variation in the timing of blood sampling between the dogs. For some, samples were collected before any treatment, while for others, sampling occurred after a variety of treatments, such as fluid therapy and antimicrobials, which could have influenced the underlying disease process and the bio-ADM concentrations. These differences in sampling timing could potentially account for lower-than-expected bio-ADM values (e.g., <22.4 pg/mL) in some dogs, which may account for a lack of significant difference in bio-ADM concentrations between dogs with sepsis and those with septic shock. Secondly, while definitive confirmation of bacterial infection was considered ideal, samples for cytology or microbiology were not obtained from dogs with suspected aspiration pneumonia. In both veterinary and human medicine, if the clinical presentation strongly indicates a bacterial infection, yet invasive sampling could compromise patient safety, other criteria can be used to support such a diagnosis [37,38]. In the cases presented here, supportive clinical signs and diagnostic imaging features, together with SIRS criteria, were used. This is reflective of clinical practice and balances potential risk of testing against a probable diagnosis necessitating immediate treatment. Thirdly, there was marked variability in the stage of sepsis present in each dog at the time of admission. Despite implementing strict inclusion criteria and applying an illness severity score for disease stratification, there was considerable variation in the clinical presentation of the dogs. The SE dogs, in particular, had a wide range of illness severity, with some having severe enough illness to require intensive care. This heterogeneity could have affected the study outcomes, making it challenging to compare the septic groups and may have contributed to the difficulties in identifying consistent patterns across the study population. Additionally, the retrospective assessment of the APPLE_fast_ scoring system poses a limitation due to the potential for subjective errors when assigning a mentation score (the only subjective value), which was made based on clinical notes. Moreover, the use of additional scoring systems, such as acute physiology and chronic health evaluation (APACHE) [39], sequential organ failure assessment (SOFA), or quick SOFA (qSOFA) [40,41,42], might have provided additional information for a more accurate assessment of disease severity between groups. Furthermore, the study did not systematically collect data on comorbidities, nor did it measure C-reactive protein concentrations in the sepsis cohorts. Comparing bio-ADM values with other commonly used biomarkers may have provided additional useful information. Lastly, the samples were freeze-stored for up to three years before analysis. However, increased bio-ADM concentrations were obtained in cases that had prolonged storage, suggesting this is not a major consideration. Additionally, in the validation of the assay, a single freeze–thaw cycle had no apparent effect on the measured bio-ADM concentrations. Human studies also suggest that bio-ADM is stable at room temperature and during prolonged storage [27].

## 5. Conclusions

In conclusion, the findings of this prospective case–control study highlight the potential value of bio-ADM as a diagnostic biomarker in dogs with sepsis when compared to healthy controls. Further studies of larger groups of dogs and assessment of bio-ADM within individual diseases should be performed to evaluate the potential value of bio-ADM further. Additionally, bio-ADM measurement in dogs with non-infectious, inflammatory conditions should be performed, as this could help in the early differentiation between septic and non-septic conditions, allowing for more appropriate initial treatment.

## Figures and Tables

**Table 1:** Summary characteristics of healthy dogs and those with sepsis and septic shock. Each group consisted of 25 dogs. Scores for APPLE_fast_ were only available for 43 dogs (SS: n = 25, SE: n = 18). APPLE_fast_, acute patient physiologic and laboratory evaluation (APPLE) fast score.

Variable	Septic Shock	Sepsis	Controls
Age (years)	6.5 (1.5–10.1)	9 (3.5–10.5) ^a^	4.5 (1.9–5.2) ^a^
Sex (Male/ Female)	15 (60%)/ 10 (40%)	14 (56%)/ 11 (44%)	12 (48%)/ 13 (52%)
Neutered status (Entire/ Neutered)	9 (36%)/ 16 (64%)	8 (32%)/ 17 (68%)	5 (20%)/ 20 (80%)
Body weight (kg)	25.4 (12.8–30.8) ^b^	17.9 (8.5–29.2) ^c^	30.4 (28.8–36.0) ^b,c^
APPLE_fast_ score	27 (23–32) ^d^	23 (18–25) ^d^	-
Outcome (non-survivors)	17 (68%) ^e^	9 (36%) ^e^	-

Note: Superscripted numbers indicate significant differences: ^a^ *p* = 0.017, ^b^ *p* = 0.009, ^c^ *p* < 0.001, ^d^ *p* = 0.007, ^e^ *p* = 0.046.

## Data Availability

The data presented in this study are available on request from the corresponding author.
